# A randomized controlled trial of a therapist-guided online intervention for depressed adults and its utility as an adjunctive to antidepressants and psychotherapy

**DOI:** 10.1186/s12888-025-06564-2

**Published:** 2025-02-11

**Authors:** Manuel Heinrich, Pavle Zagorscak, Christina Kampisiou, Johannes Bohn, Lars Schulze, Carmen Schaeuffele, Annette Brose, Christine Knaevelsrud

**Affiliations:** 1 Department of Clinical-Psychological Intervention, Freie Universität Berlin, Berlin, Germany; 2 Department of Clinical Psychology and Psychotherapy, Freie Universität Berlin, Berlin, Germany

**Keywords:** Antidepressants, Unstandardized care-as-usual (CAU), Digital intervention, Depression, Internet-based Intervention (IBI), iCBT, Randomized-controlled trial (RCT), Waitlist

## Abstract

**Background:**

Internet-based interventions (IBIs) are a low-threshold treatment for individuals with depression. However, comparisons of IBI against unstandardized care-as-usual (CAU) are scarce. Moreover, little evidence is available if IBI has an add-on effect for individuals already receiving an evidence-based treatment such as antidepressants and/or psychotherapy.

**Method:**

This parallel, two-arm RCT (1:1 allocation ratio, simple randomization) examines the effectiveness of a therapist-guided cognitive-behavioral IBI compared to unstandardized CAU in a self-selected sample of adults (≥ 18 years). Eligible individuals reported (a) mild (BDI-II score ≥ 14) to moderately severe (PHQ-9 ≤ 19) symptoms of depression, (b) no acute suicidal ideations, (c) no acute or lifetime (hypo-)mania and/or symptoms of psychosis. We assigned eligible individuals to an intervention (INT) arm or an unstandardized CAU-arm (i.e., we imposed no restrictions on what individuals were allowed to do in the 8-week waiting period). Individuals in the INT-arm got access to a 7-module CBT-based IBI. The primary endpoint is depressive symptom load 9 to 11 weeks after randomization. Secondary endpoints included anxiety, self-efficacy, and perceived social support. We report effects for the entire sample (*N* = 1899), as well as for individuals using the IBI as a stand-alone intervention (*n* = 1408) or as an add-on to antidepressants (*n* = 367), psychotherapy (*n* = 73), or antidepressants and psychotherapy (*n* = 51). Patients entered the trial with these concurrent treatments (i.e., they were not randomly assigned).

**Results:**

Concerning all randomized individuals, 62.5% of individuals in the INT-arm accessed all treatment modules within 11 weeks. Individuals assigned to the INT-arm reported significantly lower depressive symptoms (PHQ-9: − 2.5, 95% CI [− 2.9, − 2.0], *d* =  − 0.7; BDI-II: − 5.3, 95% CI [− 6.5, − 4.1], *d* =  − 0.8) and higher rates of  ≥ 50% symptom improvements (PHQ-9: 38.5% vs. 14.3%; BDI-II: 44.6% vs. 14.8%) compared to individuals assigned to the CAU-arm. Secondary outcomes also favored INT over CAU, with effect sizes ranging from |*d*|= 0.18 (social support) to 0.62 (anxiety). Rates of deterioration (PHQ-9: 4.1%; BDI-II: 3.4%) and self-reported side effects (10.5%) were low in the INT-arm. Similar patterns emerged for all strata. However, the between-arm differences failed to reach significance within the strata of individuals using the IBI as an add-on to psychotherapy.

**Conclusion:**

Our results show that providing interested adults access to the therapist-guided, cognitive-behavioral IBI under investigation is associated with improved mental health outcomes, whether individuals use the IBI as a stand-alone or add-on intervention to another evidence-based treatment. This finding aligns with available studies indicating that IBIs should be considered a low-threshold treatment option for individuals with depression.

**Trial registration:**

The trial was registered at the Deutsches Studienregister (Trial-Registriation Number/DRKS-ID: DRKS00021106, Date: 25.06.2020).

**Supplementary Information:**

The online version contains supplementary material available at 10.1186/s12888-025-06564-2.

## Introduction

Depression is the most frequent mental disorder. Recent studies indicate that 1 in 5 individuals will experience at least one depressive episode throughout their lives [1]. Subthreshold symptoms of depression, as well as symptoms of depression that fulfill the diagnostic criteria for depression, lead to impairments in individual functioning [2] and high economic costs [3, 4]. Unfortunately, depression often remains untreated [5], partly due to the limited availability of evidence-based treatment options. Guided Internet-based interventions (IBIs) have been developed to close this treatment gap as a low-threshold treatment option [6].

The most comprehensive meta-analysis conducted by Karyotaki et al. indicated that IBIs are effective in reducing symptoms of depression [6]. When compared against waitlist arms, the meta-analysis estimated the relative benefit at ─3.3 PHQ-9 points for individuals accessing an IBI [6]. When compared against CAU, the relative effect was estimated at ─1.7 PHQ-9 points [6]. Moreover, participants of IBIs for depression also report improvements in anxiety [7], rumination [8, 9], and self-reported quality of life [10]. At the same time, the database for CAU comparison is much more limited than that for waitlist comparisons, as only 7 out of 39 studies included in the aforementioned meta-analysis compared guided IBI against CAU. 4 of 7 studies favored IBI over CAU, while 3 found no significant effects [6]. Therefore, the evidence is somewhat inconclusive, and further evidence is needed. Comparisons against unstandardized CAU are especially valuable for various reasons related to treatment policy. They consider that some individuals may not receive any care although they seek treatment, while others seek additional treatment as an add-on to their current, evidence-based care. Studies like ours using unstandardized CAU for comparison allow for providing an estimate for the expected benefit of making an IBI accessible to a large group of individuals with heterogeneous treatment backgrounds. In our primary analysis, we estimate this effect for a therapist-guided, cognitive behavioral IBI in a large sample of adults with depressive symptoms. We did not restrict what individuals in the CAU-arm could do while waiting and consciously included individuals participating in other parallel treatments.

While our primary analysis does not consider which treatment individuals currently receive, our secondary analysis provides stratified effects for individuals using the IBI (1) as a stand-alone intervention or as an add-on to (2) antidepressants, (3) psychotherapy, or (4) antidepressants and psychotherapy. This analysis provides evidence on whether individuals already receiving other anti-depressive treatments still profit from IBI. Current evidence on the topic is limited.

Regarding IBIs that are used as an *add-on to psychotherapy* [11], Berger et al. found that adding an unguided IBI to psychotherapy for depression led to lower BDI-II scores compared to psychotherapy alone. However, in that trial, therapists initiated the add-on IBI treatment. Thus, effects may vary for patients who use the IBI as a self-selected add-on to their therapy. Supporting this idea, Klein et al. found only small incremental benefits (~ 1 PHQ-9 point) in patients using an IBI as a self-selected add-on to psychiatric or psychotherapeutic treatments [10]. Thus, further evidence for using an IBI as a self-selected add-on to psychotherapy is needed.

Similarly, evidence for using IBI *as an add-on to antidepressants* is limited. Mantani et al. [12] showed that individuals receiving access to an unguided IBI alongside antidepressant medication had a 2.5 points lower PHQ-9 score than individuals taking antidepressants only. Similarly, Meyer et al. [13] found that individuals using guided IBI as an add-on to medication benefited more than individuals using medication alone (~ 6 PHQ-9 points difference). However, some studies found smaller benefits of adding guided IBI to pharmacological treatments (~ 1.2 PHQ-9 points at post-assessment [10]), and others show no additional benefits [14–16]. Elaborating on the reasons for the additive effects of IBIs on pharmacological effects, some researchers argue that adding a psychological treatment addresses problem areas that mono-treatments with antidepressants do not cover sufficiently. For example, recent studies suggest that adding CBT-based treatment elements to a mono-treatment with antidepressants can lead to larger improvements in self-reported quality of life [17, 18]. Therefore, further investigations on the benefit of adding IBI to an ongoing treatment with antidepressants are needed.

## Methods

### Trial design

This study is a single-center, randomized, not-blinded, unstandardized care-as-usual (CAU) controlled trial with parallel-group assignment conducted in Germany. The trial assesses the efficacy of a therapist-guided IBI for depression in self-selected adults (age ≥ 18) from the client base of a large German health insurance provider compared to CAU. An algorithm randomly assigned subjects to the intervention arm (INT; received immediate access) or the CAU waitlist arm (received access after eight weeks) in a 1:1 ratio after the interview using simple randomization. Randomization was performed automatically for each participant using a computer-based random numbers generator supported by the host website. The clinical research team could not access, modify, or predict the random assignment. Therapists and participants were not blinded (i.e., all participants were informed about the study design before starting the screening). Individuals who returned from the CAU-arm completed the post-assessment 57 to 77 days after randomization (*Mdn* = 58 days). Therefore, we use weeks 9 to 11 after randomization as a reference for evaluating the treatment effects. All participants could use any form of concurrent support at any time.

### Participants

Participants were self-selected. Information about the program was available on the insurance company’s website. This recruitment form represents a common access point for individuals seeking mental health care in Germany. Public health care companies are trusted sources for evidence-based information on mental health and treatment options specific to their insurants. Thus, individuals suffering from depression will often seek out this information on the respective company’s website. The study and the associated IBI were advertised on all company websites that addressed depression or related topics. Anyone interested in participating could participate in the screening.

This study considers adults who entered the platform through their online insurance accounts after the 1st of July 2020 and conducted the clinical interview before the 31st of March 2022 (end of randomization). *First,* individuals completed an online questionnaire screening. We applied the following inclusion criteria throughout this online screening: (1) age ≥ 18, (2) Beck Depression Inventory-II (BDI-II) scores ≥ 14, (3) Patient-Health Questionnaire-9 (PHQ-9) scores ≤ 19, (4) no elevated suicidality (e.g., BDI-II or PHQ-9 item 9 scores ≤ 1).

**Fig. 1:**
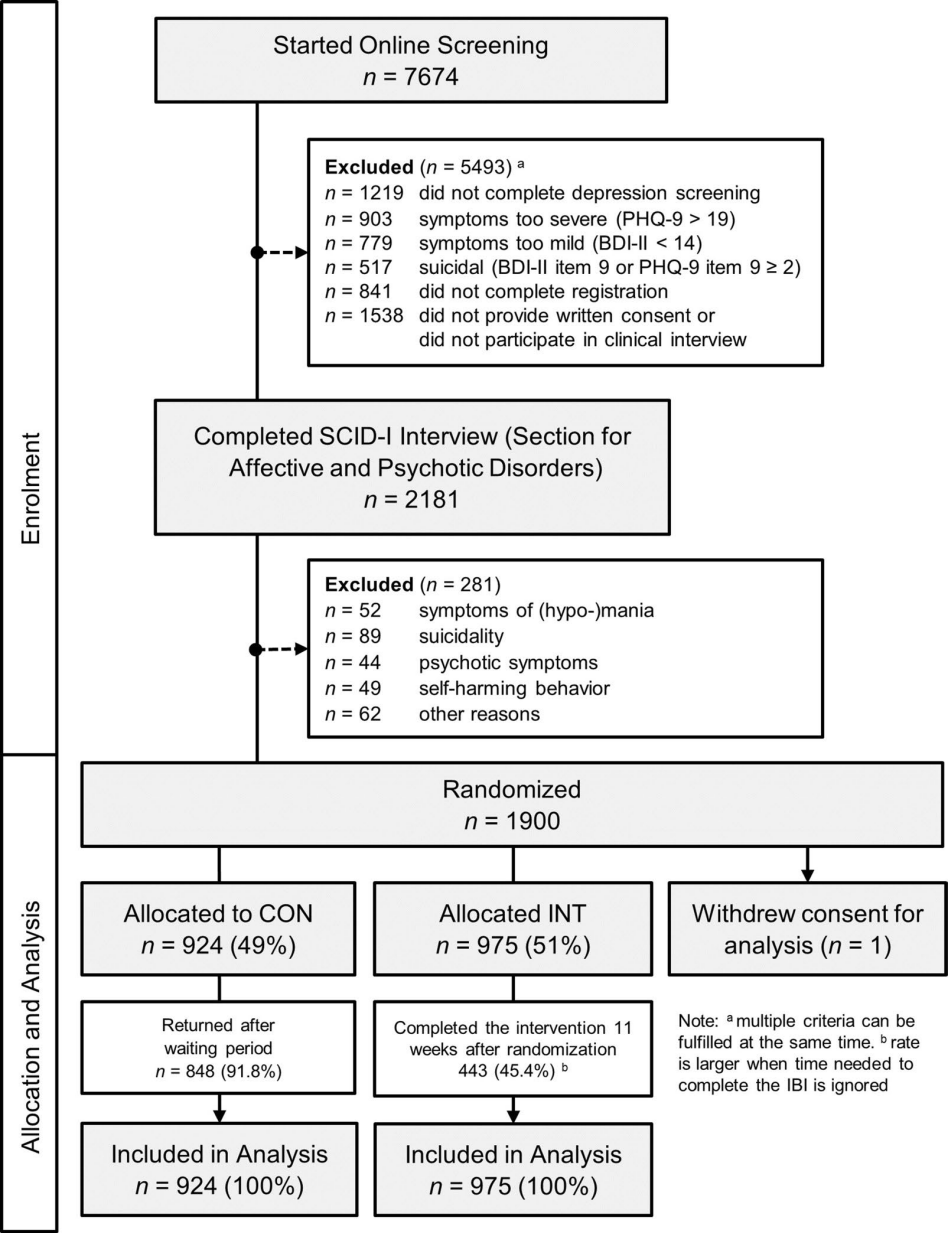
Flow-Chart

*Second,* individuals participated in a telephone-administered structured clinical-diagnostic interview (SCID-I, section A on affective disorders, and section B on psychotic symptoms; [19]) conducted by trained interviewers. We applied the following exclusion criteria throughout the interview: (1) *current/lifetime symptoms of (hypo-)mania* (i.e., individuals fulfilling the diagnostic criteria for a current or lifetime diagnosis of [hypo-]mania) *and/or psychosis* (i.e., individuals reporting lifetime psychotic symptoms); (2) *elevated risk of suicidality.* Individuals reporting self-harm were also excluded due to the associated heightened suicide risk [20]. We excluded some individuals for other reasons, such as language barriers (i.e., individuals with insufficient German language skills to complete the interview in German) or individuals who considered the treatment inappropriate for their situation after the interviewer explained the intervention. Note that a current MDE diagnosis was not considered an inclusion criterion. Please see Fig. 1 for the flow chart.

### Intervention and control

The guided IBI encompassed seven consecutive modules on structured writing, behavioral activation, and cognitive restructuring (see Table 1). 11 licensed psychotherapists trained in the intervention program were equipped with a manual that provided written guidance. Research staff reviewed each therapist’s first two module-specific feedbacks to ensure manual adherence. All therapists used standardized templates with text modules to structure the feedback and adapted these templates to the participants’ progress. They assessed perceived burdens and guided the optimal use of the intervention modules.

**Table 1:** Description of treatment modules

General Structure of the Treatment Modules
Each module followed the same structure: Participants (1) accessed the written feedback in which the therapist commented on progress and work on the previous modules, (2) received multimedia psychoeducation on a module-specific topic, and (3) received instructions on how to work on the new task. Participants received feedback if they completed a structured writing task (Modules 1, 2, 7) or had seven days to work on the module content (Modules 3, 4, 5, 6). Inactive participants received standardized reminder emails
Module 1: Understanding Depression I
• Psychoeducation: prevalence of depression and core symptoms (anhedonia and sadness) • Task: structured writing (~ 45 min). Participants write a letter describing their initial experience with depression, with a specific focus on their emotional, cognitive, and physical encounters during the episode. Furthermore, the task encourages participants to recall effective strategies and resources they utilized to cope with distress
Module 2: Understanding Depression II
• Psychoeducation: secondary symptoms of depression (focus on somatic and cognitive symptoms) • Task: structured writing (~ 45 min). Participants write about current distress (e.g., at work, social interactions, or other life contexts). Participants are encouraged to describe their depressive feelings, cognitions, and physical reactions in response to a prototypical distressing situation they recently experienced. Participants explore which personal and social resources they have available to deal with their current symptoms. Participants define treatment goals they wish to achieve during participation
Module 3: Behavioral Activation I
• Psychoeducation: Association between Mood and Activity (downward Spiral of depression) • Task: behavioral activation (~ 15 to 25 min., daily). Participants collect their preferred pleasant activities in a list. Participants use an online day planner with a special emphasis on planning pleasant activities
Module 4: Behavioral Activation II
• Psychoeducation: Deepen the understanding of the link between mood and activity. Participants learn the concept of coping planning to deal with unexpected barriers that hamper goal attainment • Task: behavioral activation (~ 15 to 25 min., daily). Participants continue to work with the online day planner. In contrast to module 3, participants are now encouraged to write coping plans to realize scheduled pleasant (and unpleasant) activities
Module 5: Cognitive Restructuring 1
• Psychoeducation: Effects of dysfunctional cognitions on mood (emphasis on Beck’s cognitive biases) • Task: multimedia bias training (~ 30 min., daily). Participants watch videos (or read texts or listen to audio recordings) describing open-ended (socially) ambivalent situations, such as the scenario where “friends of yours met at a bar and did not invite you.” First, participants should develop a vivid mentalization of this situation. Following this, the platform presents participants with potential outcomes, representing different reasons why they may not have been invited. Participants then select the outcome that aligns most closely with their thinking. Subsequently, the actual outcome is revealed. Upon encountering multiple situations, the platform provides participants with automated feedback highlighting the frequency of their selection of negative or positive interpretations. This feedback illustrates various cognitive biases (e.g., “black-white-thinking”) and their impact on well-being
Module 6: Cognitive Restructuring II
• Psychoeducation focuses on deepening knowledge of the impact of dysfunctional cognitions on mood • Task: multimedia bias training (~ 30 min, daily): In this module, participants are additionally asked to develop three different outcomes to the presented ambivalent situations (instead of being provided possible outcomes by the intervention) and rate them in terms of their likelihood and consistency with their typical way of thinking. Participants receive access to an online thought protocol. Participants log situations, thoughts, and alternative thoughts about stressful events over the next week (~ 15 min., daily)
Module 7: Relapse Prevention
• Psychoeducation: recognizing personal warning signs and establishing routines that help to maintain improvements • Task: structured writing (~ 45 min). Participants write a letter to their former selves about differences in well-being before and after the intervention, personal warning signs, and useful strategies they learned and plan to use after the intervention. In addition, the tasks encourage participants to offer their former selves an optimistic perspective of the future

All randomized individuals received a welcome message in which the therapists introduced themselves. In the following, therapists provided written feedback whenever a participant completed a module. For modules 1, 2, and 7 (i.e., the structured writing tasks), completion was defined as participants submitting a text. We considered modules 3 through 6 (i.e., the behavioral activation and cognitive restructuring tasks) “completed” once participants had seven days to engage with the material. In these modules, feedback was provided regardless of the extent of participants’ engagement. For example, participants who did not complete any activities within seven days received written feedback addressing typical barriers and encouraging completion of subsequent activities. Therapists sent feedback within two business days of task completion.

Participants could write additional messages to their therapists on the platform. Therapists answered requests via a separate message, within the next feedback text, or via telephone, but only if the therapists could not answer the request via a written message.

Participants reporting suicidality in module-wise PHQ-9 ratings received an automated message outlining possible actions. Participants who reported a PHQ-9 item 9 score ≥ 2 were contacted via telephone by the therapist—a trained clinician supervised crisis interventions.

The control arm represents unstandardized CAU; this means that some individuals may do nothing to cope with their mental health problems, while others may take up other approaches to mental health care. There were no restrictions on what individuals were allowed to do while waiting.

### Outcome measures

The primary outcome was depressive symptom load measured with the PHQ-9 (range: 0–27; severity classification: 0–5 minimal, 6–9 mild, 10–14 moderate, 15–19 moderately severe, and 20–27 severe) and BDI-II (range: 0–63; severity classification: 0–13 minimal, 14–19 mild, 20–28 moderate, and 29–63 severe). Secondary outcomes were anxiety (*Generalized Anxiety Disorder* Scale, GAD; range 0–21; severity classification: 0–4 minimal, 5–9: mild, 10–14 moderate, 15–21 severe [21]), cognitive biases (*Cognitive Styles Assessment*, COSTA; range: 0–72; [22]), and restrictions in participation (*Index for Measurement of Impairments in Participation;* IMET, range: 0–90; [23]), perceived social support (range: 8 − 32) and support-seeking behavior (range: 5 − 20; *Berlin Social Support Scale,* BSSS, [24]), self-efficacy (*Generalized Self-Efficacy Scale,* GSE, range 10–40; [25]), and quality of life (EUROHIS-QOL, [26]). Lower BDI-II, PHQ-9, GAD-7, COSTA, and IMET scores are favorable. The opposite is true for the BSSS, GSE, and EUROHIS-QOL.

We assessed acceptability through treatment satisfaction, the likelihood of recommending the IBI, perceived helpfulness, and self-reported side effects, including their impact on well-being.

#### Definition of concurrent treatment-status

Information collected in the telephone-administered diagnostic interview formed the basis for classifying the concurrent treatment status at randomization. Participants entered the trial while participating in these concurrent treatments. Consequently, the secondary treatments were not assigned randomly.

##### Concurrent antidepressants (MED)

Interviewers asked participants if they were currently taking medication that affects their mood and about the brand name of the medication. Individuals were classified as taking antidepressants if they reported taking SSRIs (*n* = 239, 57.2%)*,* SNRIs (*n* = 65, 15.1%), Tetra- or Tricyclic antidepressants (*n* = 64, 15.3%), or other antidepressants such as Agomelatine, Bupropion, Mirtazapine, MAOs, Trazodone, and Tianeptine (*n* = 104, 24.9%).

##### Psychotherapy (PSY)

Interviewers asked individuals if they were currently receiving psychotherapy. If they answered ‘yes,’ interviewers checked that participants received evidence-based psychotherapy (e.g., cognitive-behavioral, psychodynamic, psychoanalytic, or systemic psychotherapy) instead of other forms of psychosocial support (e.g., irregular appointments with psychiatrists). If participants could not name the psychotherapeutic approach, we included individuals who reported that a licensed psychotherapist provided their treatment. We classified participants who reported seeing their therapist at least every two weeks as receiving psychotherapy.

##### No antidepressants or psychotherapy (IBI-ONLY)

We considered all individuals not receiving medication and/or current psychotherapy as members of the IBI-ONLY stratum.

### Statistical analysis

In line with previous work [8, 9], we consider (1) a module to be “completed” when the therapist has provided feedback on that module and (2) the intervention to be “completed” as soon as participants had access to the entirety of the treatment material. We report completion rates focusing on 11 weeks after randomization, which allows for a fair evaluation of the expected adherence within the time individuals in the CAU-arm waited for access.

We also report dropout rates. We consider an individual “dropped out” if the participant had not gained access to all treatment modules within the first 11 weeks and did not work on further modules than those completed within 11 weeks after randomization.

We computed within-arm changes as average change scores. The effect of providing individuals access to the IBI (e.g., the between-arm effect) was determined using a linear regression adjusted for baseline symptom severity (ANCOVA model; INT = 1, CAU = 0). Bonferroni-based alpha-error correction was performed for each stratum separately. A between-arm difference with *p* < 0.025 (0.05/2) was considered significant concerning the main outcomes. Concerning the secondary outcome measures, a between-arm difference with *p* < 0.006 (0.05/8) was considered significant.

We report unstandardized and standardized within- and between-arm differences using the baseline standard deviation of all randomized individuals as a standardizer with bootstrapped 95% CIs (200 bootstrap draws per imputed dataset). The same standardizer was employed for all strata to ensure that *d* is comparable across strata. Since secondary treatments were not assigned randomly, we refrained from between-strata comparisons.

We report absolute risk differences (ARD) for meaningful improvements (≥ 50% symptom improvement, [27]) and deteriorations (symptom increases of 5 PHQ-9/10 BDI-II points).

The trial focuses on the expected benefits of providing interested individuals access to an IBI as indicated by the average between-arm difference in symptom severity from week 9 to 11 after randomization irrespective of treatment usage and add-on-treatments (intention-to-treat effect: expected health benefit under non-perfect adherence in a real-world clinical application of the IBI). We used multiple imputations implemented in *mice* (50 imputations, 30 iterations, and predictive mean matching on the level of sum scores [28]) for all imputation steps. We conducted the imputation arm-wise (CAU and INT). We constructed a conservative estimator as follows.

First, we imputed individuals assigned to INT who did not start to work with the intervention or only completed a minimal number of modules (i.e., no more than the first two writing tasks) within 11 weeks after randomization together with individuals from the CAU-arm (*n* = 105). This approach assumes that expressive writing yields only placebo- or non-specific effects [29]. This conservative strategy represents the assumption that individuals receiving minimal intervention show a similar symptom course to those assigned to the CAU-arm. Second, within the remaining individuals in the INT-arm (e.g., those who at least started with module 3 within 11 weeks after randomization, *n* = 870), we only considered the post-assessment provided 11 weeks after randomization as “valid” (*n* = 420). If individuals did not provide the post-assessment but a PHQ-9 pre-module rating in weeks 9 to 11 after randomization, this PHQ-9 score was used as a proxy for the remaining outcomes (*n* = 296). The remaining cases (*n* = 154) did not provide a valid post-assessment or an appropriate PHQ-9 proxy. The imputation model included the number of modules, age, sex, baseline scores, and dummies for the add-on treatments as auxiliary variables.

In a comprehensive sensitivity analysis, we increased the imputed PHQ-9 and BDI-II scores of individuals in the INT-arm not already imputed with the CAU-arm by 10%, 20%, 30%, 40%, and 50% and repeated the analysis (delta-adjustment). This approach mimics an NMAR scenario in which our imputations underestimate the symptom load of individuals within the INT-arm, which could lead to overestimating the between-arm effect. We provide the results in supplemental Tables.

Individuals who completed the post-assessment provided data on acceptability and side effects. Individuals gained access to the post-assessment if they completed all modules or actively decided to leave the IBI. To avoid overestimating satisfaction, the imputation models for side effects considered only information individuals provided until they either finished or left the IBI (i.e., while-on-treatment symptom change, baseline scores, and the number of completed modules).

## Results

### Participants

The *N* = 1899 randomized individuals (IBI-ONLY: *n* = 1408, 74.1%; MED: *n* = 367, 19.3%; MED + PSY: *n* = 51, 2.7%; PSY: *n* = 73, 3.8%) were on average 39.0 years old (*SD* = 11.6). Overall, *n* = 1233 (64.9%) were female, and *n* = 937 (49.3%) individuals fulfilled the diagnostic criteria for current MDE. Table 2 provides further sample characteristics.

**Table 2:** Sample characteristics

Strata	OVERALL		S: MED		S: PSY		S: MED + PSY		S: IBI-ONLY	
Arm	CAU	INT	CAU	INT	CAU	INT	CAU	INT	CAU	INT
*N*	924	975	177	190	42	31	25	26	680	728
Female, *n* (%)	603 (65.3%)	630 (64.6%)	101 (57.1%)	114 (60.0%)	28 (66.7%)	23 (74.2%)	19 (76%)	17 (65.4%)	455 (66.9%)	476 (65.4%)
Age, *M* (*SD*)	39.4 (11.8)	38.7 (11.3)	42.7 (11.3)	42.4 (11.6)	39.2 (12.1)	38.5 (9.3)	44.6 (9.3)	43.9 (13.6)	38.4 (11.8)	37.5 (11.0)
Education, *n* (%)										
No Certificate	1 (0.1%)	0 (0.0%)	0 (0.0%)	0 (0.0%)	0 (0.0%)	0 (0.0%)	0 (0.0%)	0 (0.0%)	1 (0.1%)	0 (0.0%)
Lower Secondary	21 (2.3%)	15 (1.5%)	8 (4.5%)	3 (1.6%)	1 (2.4%)	0 (0.0%)	1 (4.0%)	1 (3.8%)	11 (1.6%)	11 (1.5%)
Secondary School	118 (12.8%)	120 (12.3%)	36 (20.3%)	38 (20.0%)	5 (11.9%)	3 (9.7%)	8 (32.0%)	1 (3.8%)	69 (10.1%)	78 (10.7%)
Trade school	214 (23.2%)	248 (25.4%)	40 (22.6%)	50 (26.3%)	5 (11.9%)	7 (22.6%)	8 (32.0%)	5 (19.2%)	161 (23.7%)	186 (25.5%)
College	99 (10.7%)	114 (11.7%)	18 (10.2%)	29 (15.3%)	9 (21.4%)	0 (0.0%)	2 (8.0%)	3 (11.5%)	70 (10.3%)	82 (11.3%)
University	471 (51.0%)	478 (49.0%)	75 (42.4%)	70 (36.8%)	22 (52.4%)	21 (67.7%)	6 (24%)	16 (61.5%)	368 (54.1%)	371 (51.0%)
Marital status, *n* (%)										
Single	468 (50.6%)	495 (50.8%)	75 (42.4%)	74 (38.9%)	20 (47.6%)	16 (51.6%)	9 (36.0%)	10 (38.5%)	364 (53.5%)	395 (54.3%)
Partnership/Married	379 (41%)	403 (41.3%)	83 (46.9%)	99 (52.1%)	20 (47.6%)	11 (35.5%)	12 (48.0%)	13 (50.0%)	264 (38.8%)	280 (38.5%)
Divorced	73 (7.9%)	64 (6.6%)	19 (10.7%)	15 (7.9%)	2 (4.8%)	4 (12.9%)	4 (16.0%)	3 (11.5%)	48 (7.1%)	42 (5.8%)
Widowed	4 (0.4%)	13 (1.3%)	0 (0.0%)	2 (1.1%)	0 (0.0%)	0 (0.0%)	0 (0.0%)	0 (0.0%)	4 (0.6%)	11 (1.5%)
BDI-II severity, *n* (%)										
Mild (14–19)	211 (22.8%)	222 (22.8%)	37 (20.9%)	32 (16.8%)	12 (28.6%)	4 (12.9%)	5 (20.0%)	5 (19.2%)	157 (23.1%)	181 (24.9%)
Moderate (20–28)	428 (46.3%)	461 (47.3%)	74 (41.8%)	77 (40.5%)	16 (38.1%)	13 (41.9%)	10 (40.0%)	13 (50.0%)	328 (48.2%)	358 (49.2%)
Severe (29–63)	285 (30.8%)	292 (29.9%)	66 (37.3%)	81 (42.6%)	14 (33.3%)	14 (45.2%)	10 (40.0%)	8 (30.8%)	195 (28.7%)	189 (26.0%)
PHQ-9 severity, *n* (%)										
Minimal (0–4)	2 (0.2%)	5 (0.5%)	0 (0.0%)	2 (1.1%)	0 (0.0%)	0 (0.0%)	0 (0.0%)	0 (0.0%)	2 (0.3%)	3 (0.4%)
Mild (5–9)	166 (18.0%)	184 (18.9%)	19 (10.7%)	24 (12.6%)	10 (23.8%)	8 (25.8%)	6 (24.0%)	6 (23.1%)	131 (19.3%)	146 (20.1%)
Moderate (10–14)	418 (45.2%)	465 (47.7%)	75 (42.4%)	86 (45.3%)	18 (42.9%)	9 (29.0%)	8 (32.0%)	9 (34.6%)	317 (46.6%)	361 (49.6%)
Moderate-Severe (15–19)	338 (36.6%)	321 (32.9%)	83 (46.9%)	78 (41.1%)	14 (33.3%)	14 (45.2%)	11 (44.0%)	11 (42.3%)	230 (33.8%)	218 (29.9%)
SCID diagnosis cur. MDE, *n* (%)	447 (48.4%)	490 (50.3%)	98 (55.4%)	107 (56.3%)	25 (59.5%)	16 (51.6%)	14 (56.0%)	13 (50.0%)	310 (45.6%)	354 (48.6%)

The table presents sample characteristics for all randomized individuals (OVERALL), as well as for each stratum: individuals taking medication (S: MED), receiving psychotherapy (S: PSY), receiving both psychotherapy and medication (S: MED + PSY), and those receiving neither psychotherapy nor medication (S: IBI-ONLY)

*CAU* unstandardized Care-as-Usual arm, *INT* Intervention arm

### Adherence

Overall, *n* = 609 (62.5%) of individuals in the INT-arm completed at least six modules within 11 weeks. The numbers across strata are: MED: *n* = 119 (62.6%); PSY: *n* = 13 (41.9%); MED + PSY: *n* = 14 (53.8%), IBI-ONLY: *n* = 463 (63.6%; see Table 3 for module-wise rates). 195 of 975 (20%) individuals of the INT-arm dropped out within 11 weeks after randomization (i.e., had not gained access to all treatment modules within the first 11 weeks and did not work on further modules than those completed within 11 weeks after randomization). The numbers across strata are as follows: MED: *n* = 43 (22.6%); PSY: *n* = 4 (12.9%); MED + PSY: *n* = 6 (23.1%); IBI-ONLY: *n* = 142 (19.5%).

**Table 3:** Completion rates within individuals assigned to the INT-arm

	OVERALL	S: MED	S: PSY	S: MED + PSY	S: IBI-ONLY
Completed …	*n* (%)	*n* (%)	*n* (%)	*n* (%)	*n* (%)
… no module	60 (6.2)	15 (7.9)	2 (6.5)	2 (7.7)	41 (5.6)
… up to M1	37 (3.8)	9 (4.7)	3 (9.7)	0 (0)	25 (3.4)
… up to M2	16 (1.6)	3 (1.6)	0 (0)	1 (3.8)	12 (1.6)
… up to M3	54 (5.5)	11 (5.8)	2 (6.5)	3 (11.5)	38 (5.2)
… up to M4	67 (6.9)	13 (6.8)	4 (12.9)	2 (7.7)	48 (6.6)
… up to M5	132 (13.5)	20 (10.5)	7 (22.6)	4 (15.4)	101 (13.9)
… up to M6	166 (17)	41 (21.6)	5 (16.1)	5 (19.2)	115 (15.8)
… up to M7	443 (45.4)	78 (41.1)	8 (25.8)	9 (34.6)	348 (47.8)
Compl. Rate	62.5	62.6	41.9	53.8	63.6
95% CI for %	(59.3, 65.6)	(55.3, 69.5)	(24.5, 60.9)	(33.4, 73.4)	(60.0, 67.1)

Number of Modules participants assigned to the INT-arm completed within 11 weeks after randomization. The completion rate was defined as gaining access to all treatment modules (i.e., completing at least module 6). Completion rates are provided for all randomized individuals (OVERALL) and each stratum: individuals taking medication (S: MED), receiving psychotherapy (S: PSY), receiving both psychotherapy and medication (S: MED + PSY), and those receiving neither psychotherapy nor medication (S: IBI-ONLY)

### Within- and between-arm effects

Table 4 shows within-arm changes. Table 5 summarizes between-arm effects. Table 6 shows rates of meaningful improvements and deterioration. We report results for the entire sample, as well as for each stratum. If not stated otherwise, all results were derived using the imputed data set. That is, between-arm effects represent the effect of providing individuals access to an IBI, as the imputation strategy considers imperfect adherence.

**Table 4:** Within-arm changes

Scale	Strata	Within-Arm Change—CAU			Within-Arm Change—INT		
		PRE, *M* (*SD*)	∆ (95% CI)	*d* (95% CI)	PRE, *M* (*SD*)	∆ (95% CI)	*d* (95% CI)
PHQ-9	OVERALL	13.0 (3.5)	− 2.1 (− 2.4, − 1.8)	− 0.61 (− 0.69, − 0.53)	12.8 (3.5)	− 4.5 (− 4.8, − 4.2)	− 1.28 (− 1.38, − 1.19)
	S: MED	13.7 (3.3)	− 2.3 (− 2.9, − 1.6)	− 0.65 (− 0.84, − 0.46)	13.6 (3.4)	− 4.9 (− 5.7, − 4.1)	− 1.41 (− 1.63, − 1.18)
	S: PSY	13.2 (3.7)	− 3.0 (− 4.3, − 1.6)	− 0.84 (− 1.22, − 0.46)	13.0 (4.1)	− 3.7 (− 6.0, − 1.4)	− 1.05 (− 1.63, − 0.45)
	S: MED + PSY	13.7 (3.6)	− 2.6 (− 4.8, − 0.4)	− 0.74 (− 1.34, − 0.18)	13.1 (3.9)	− 6.1 (− 8.2, − 3.9)	− 1.74 (− 2.26, − 1.13)
	S: IBI-ONLY	12.7 (3.5)	− 2.0 (− 2.4, − 1.7)	− 0.58 (− 0.67, − 0.48)	12.5 (3.5)	− 4.4 (− 4.7, − 4.0)	− 1.24 (− 1.35, − 1.14)
BDI-II	OVERALL	25.2 (6.7)	− 4.8 (− 5.4, − 4.3)	− 0.73 (− 0.81, − 0.66)	25.1 (6.5)	− 10.1 (− 11.3, − 9.0)	− 1.54 (− 1.72, − 1.38)
	S: MED	26.0 (7.0)	− 5.0 (− 6.2, − 3.7)	− 0.75 (− 0.93, − 0.56)	26.9 (7.0)	− 11.1 (− 13.1, − 9.1)	− 1.69 (− 1.97, − 1.39)
	S: PSY	24.5 (6.2)	− 6.2 (− 8.8, − 3.5)	− 0.93 (− 1.32, − 0.56)	26.2 (5.7)	− 8.0 (− 13.9, − 2.1)	− 1.21 (− 1.96, − 0.39)
	S: MED + PSY	26.9 (7.9)	− 5.9 (− 10.2, − 1.5)	− 0.89 (− 1.53, − 0.32)	25.8 (6.9)	− 14.1 (− 19.5, − 8.6)	− 2.13 (− 2.84, − 1.39)
	S: IBI-ONLY	24.9 (6.6)	− 4.7 (− 5.3, − 4.1)	− 0.71 (− 0.80, − 0.62)	24.6 (6.3)	− 9.8 (− 11.0, − 8.6)	− 1.49 (− 1.67, − 1.32)
GAD-7	OVERALL	11.0 (3.9)	− 1.7 (− 2.0, − 1.5)	− 0.46 (− 0.53, − 0.39)	10.8 (3.8)	− 4.0 (− 4.5, − 3.5)	− 1.05 (− 1.18, − 0.92)
	S: MED	11.2 (3.8)	− 2.0 (− 2.6, − 1.4)	− 0.53 (− 0.68, − 0.37)	11.1 (4.0)	− 4.6 (− 5.4, − 3.7)	− 1.19 (− 1.40, − 0.97)
	S: PSY	11.4 (3.4)	− 2.7 (− 4.0, − 1.4)	− 0.70 (− 1.02, − 0.37)	11.1 (3.1)	− 3.3 (− 5.6, − 1.0)	− 0.86 (− 1.39, − 0.30)
	S: MED + PSY	10.7 (4.0)	− 1.2 (− 3.3, 0.9)	− 0.32 (− 0.85, 0.16)	10.1 (4.4)	− 5.8 (− 8.2, − 3.3)	− 1.51 (− 2.06, − 0.91)
	S: IBI-ONLY	10.9 (3.9)	− 1.6 (− 2.0, − 1.3)	− 0.43 (− 0.51, − 0.35)	10.7 (3.7)	− 3.8 (− 4.4, − 3.3)	− 1.00 (− 1.14, − 0.87)
IMET	OVERALL	42.1 (15.4)	− 3.7 (− 4.7, − 2.7)	− 0.24 (− 0.31, − 0.18)	42.1 (14.9)	− 11.8 (− 14.3, − 9.3)	− 0.78 (− 0.93, − 0.62)
	S: MED	47.5 (16.7)	− 5.1 (− 7.5, − 2.7)	− 0.34 (− 0.49, − 0.18)	48.2 (14.3)	− 15.7 (− 19.6, − 11.8)	− 1.04 (− 1.28, − 0.79)
	S: PSY	44.0 (13.2)	− 7.3 (− 12.1, − 2.5)	− 0.48 (− 0.78, − 0.18)	42.3 (13.6)	− 4.9 (− 14.9, 5.1)	− 0.32 (− 0.86, 0.26)
	S: MED + PSY	45.2 (14.9)	− 2.6 (− 8.7, 3.5)	− 0.17 (− 0.56, 0.20)	43.8 (14.3)	− 22.5 (− 33.7, − 11.4)	− 1.49 (− 2.11, − 0.84)
	S: IBI-ONLY	40.4 (14.8)	− 3.1 (− 4.2, − 2.0)	− 0.21 (− 0.28, − 0.13)	40.5 (14.8)	− 10.7 (− 13.3, − 8.1)	− 0.70 (− 0.87, − 0.54)
COSTA	OVERALL	32.6 (10.2)	− 1.5 (− 2.0, − 1.0)	− 0.15 (− 0.20, − 0.10)	32.1 (10.3)	− 7.2 (− 8.7, − 5.8)	− 0.70 (− 0.84, − 0.58)
	S: MED	31.3 (10.9)	− 1.7 (− 2.8, − 0.6)	− 0.17 (− 0.28, − 0.06)	32.6 (11.2)	− 9.7 (− 11.6, − 7.7)	− 0.94 (− 1.13, − 0.76)
	S: PSY	31.8 (9.8)	− 0.8 (− 3.7, 2.1)	− 0.08 (− 0.36, 0.19)	32.6 (10.5)	− 5.4 (− 11.4, 0.6)	− 0.53 (− 1.07, − 0.01)
	S: MED + PSY	32.7 (11.0)	− 0.8 (− 4.3, 2.7)	− 0.08 (− 0.41, 0.22)	31.2 (13.4)	− 14.3 (− 20.7, − 7.9)	− 1.39 (− 1.95, − 0.86)
	S: IBI-ONLY	32.9 (10.0)	− 1.5 (− 2.1, − 1.0)	− 0.15 (− 0.21, − 0.09)	32.0 (9.9)	− 6.4 (− 8.0, − 4.8)	− 0.62 (− 0.78, − 0.49)
PHQ-S	OVERALL	9.3 (3.3)	− 1.1 (− 1.3, − 0.9)	− 0.34 (− 0.40, − 0.27)	9.3 (3.2)	− 2.4 (− 2.9, − 1.9)	− 0.73 (− 0.86, − 0.59)
	S: MED	9.5 (3.3)	− 1.3 (− 1.7, − 0.8)	− 0.38 (− 0.52, − 0.24)	10.0 (3.3)	− 3.4 (− 4.2, − 2.7)	− 1.05 (− 1.29, − 0.84)
	S: PSY	9.2 (3.5)	− 1.1 (− 2.3, 0.0)	− 0.35 (− 0.68, − 0.01)	10.0 (3.5)	− 2.4 (− 4.6, − 0.1)	− 0.73 (− 1.35, − 0.11)
	S: MED + PSY	10.2 (3.2)	− 1.5 (− 3.0, − 0.0)	− 0.46 (− 0.89, − 0.05)	9.5 (2.8)	− 3.5 (− 5.8, − 1.2)	− 1.07 (− 1.68, − 0.47)
	S: IBI-ONLY	9.2 (3.3)	− 1.0 (− 1.3, − 0.8)	− 0.32 (− 0.40, − 0.25)	9.0 (3.2)	− 2.1 (− 2.5, − 1.6)	− 0.64 (− 0.77, − 0.50)
GSE	OVERALL	24.3 (4.8)	0.8 (0.5, 1.0)	0.16 (0.11, 0.21)	24.0 (5.0)	2.9 (2.2, 3.6)	0.59 (0.46, 0.72)
	S: MED	23.6 (4.5)	0.9 (0.3, 1.5)	0.18 (0.06, 0.30)	23.2 (5.0)	3.5 (2.5, 4.5)	0.72 (0.53, 0.92)
	S: PSY	23.9 (4.8)	1.0 (− 0.2, 2.2)	0.20 (− 0.03, 0.44)	23.1 (5.2)	2.2 (− 0.9, 5.3)	0.45 (− 0.07, 1.00)
	S: MED + PSY	23.1 (5.6)	1.1 (− 0.6, 2.8)	0.23 (− 0.06, 0.59)	23.8 (6.0)	4.5 (1.1, 8.0)	0.93 (0.32, 1.54)
	S: IBI-ONLY	24.6 (4.8)	0.7 (0.5, 1.0)	0.15 (0.10, 0.21)	24.3 (4.9)	2.7 (2.0, 3.4)	0.55 (0.41, 0.68)
EURO-QOL	OVERALL	24.1 (4.0)	1.1 (0.9, 1.4)	0.28 (0.22, 0.34)	24.0 (4.2)	3.3 (2.7, 3.9)	0.80 (0.67, 0.95)
	S: MED	23.4 (4.1)	1.3 (0.7, 1.9)	0.32 (0.17, 0.46)	22.9 (4.0)	4.2 (3.2, 5.2)	1.02 (0.80, 1.28)
	S: PSY	23.1 (3.6)	2.1 (0.9, 3.2)	0.51 (0.25, 0.78)	23.2 (4.2)	2.2 (− 0.0, 4.4)	0.54 (0.02, 1.00)
	S: MED + PSY	23.8 (3.9)	1.1 (− 0.6, 2.8)	0.27 (− 0.11, 0.68)	24.0 (3.4)	7.3 (4.6, 10.1)	1.80 (1.18, 2.35)
	S: IBI-ONLY	24.4 (3.9)	1.1 (0.8, 1.3)	0.26 (0.19, 0.32)	24.3 (4.2)	2.9 (2.3, 3.6)	0.72 (0.58, 0.88)
BSSS-s	OVERALL	11.4 (3.6)	0.4 (0.2, 0.6)	0.11 (0.06, 0.16)	11.6 (3.6)	1.6 (1.1, 2.0)	0.44 (0.32, 0.55)
	S: MED	11.6 (3.5)	0.2 (− 0.3, 0.6)	0.04 (− 0.08, 0.16)	11.4 (3.6)	1.7 (1.1, 2.4)	0.47 (0.30, 0.65)
	S: PSY	11.8 (3.7)	0.8 (− 0.1, 1.6)	0.21 (− 0.01, 0.44)	11.9 (3.9)	0.3 (− 1.7, 2.3)	0.08 (− 0.40, 0.54)
	S: MED + PSY	11.0 (4.0)	0.9 (− 0.1, 2.0)	0.26 (− 0.01, 0.54)	12.7 (4.3)	2.9 (1.0, 4.8)	0.80 (0.35, 1.26)
	S: IBI-ONLY	11.4 (3.6)	0.4 (0.2, 0.6)	0.11 (0.06, 0.17)	11.6 (3.5)	1.6 (1.1, 2.0)	0.44 (0.31, 0.55)
BSSS-e	OVERALL	25.0 (5.4)	0.9 (0.6, 1.1)	0.16 (0.12, 0.21)	25.3 (5.4)	1.8 (1.1, 2.4)	0.33 (0.22, 0.43)
	S: MED	25.4 (5.3)	1.1 (0.5, 1.7)	0.21 (0.10, 0.31)	25.1 (5.6)	2.0 (1.1, 2.9)	0.37 (0.20, 0.53)
	S: PSY	25.5 (5.6)	− 0.1 (− 1.2, 1.0)	− 0.02 (− 0.22, 0.17)	24.7 (5.4)	1.1 (− 1.3, 3.5)	0.20 (− 0.21, 0.58)
	S: MED + PSY	25.5 (5.4)	1.5 (− 0.2, 3.2)	0.28 (− 0.01, 0.58)	28.2 (4.0)	1.1 (− 1.0, 3.1)	0.20 (− 0.15, 0.51)
	S: IBI-ONLY	24.9 (5.4)	0.9 (0.6, 1.1)	0.16 (0.11, 0.21)	25.3 (5.3)	1.8 (1.1, 2.5)	0.33 (0.21, 0.45)

Within-arm changes are presented for all randomized individuals (OVERALL) and each stratum: individuals taking medication (S: MED), receiving psychotherapy (S: PSY), receiving both psychotherapy and medication (S: MED + PSY), and those not receiving either psychotherapy or medication (S: IBI-ONLY). Positive within-arm differences indicate more favorable outcomes for GSE, EUROHIS-QOL, and BSSS. For all other outcomes, negative differences indicate improvements. ∆ = unstandardized within-arm changes. d = Cohen’s *d*-like effect measure

*BSSS-e, BSSS-s* Berlin Social Support Scale, emotional support and support-seeking behavior, *BDI-II* Beck-Depression-Inventory-II, *COSTA* Cognitive Style Assessment, *EUROHIS-QOL* Quality of Life Assessment, *GAD-7* Generalized Anxiety Scale, *GSE* General Self-Efficacy, *IMET* Index for Measurement of Impairments in Participation, *PHQ-S* PHQ Stress, *PHQ-9* Patient Health-Questionnaire 9

**Table 5:** Between-arm effects

Scale	Stratum	∆ (95% CI)	*p*	*d* (95% CI)
PHQ-9	OVERALL	− 2.5 (− 2.9, − 2.0)	< .001	− 0.70 (− 0.82, − 0.58)
	S: MED	− 2.7 (− 3.7, − 1.7)	< .001	− 0.77 (− 1.04, − 0.50)
	S: PSY	− 0.8 (− 3.1, 1.5)	.478^NS^	− 0.23 (− 0.89, 0.40)
	S: MED + PSY	− 3.8 (− 6.6, − 1.1)	.008	− 1.09 (− 1.78, − 0.43)
	S: IBI-ONLY	− 2.4 (− 2.9, − 2.0)	< .001	− 0.69 (− 0.82, − 0.56)
BDI-II	OVERALL	− 5.3 (− 6.5, − 4.1)	< .001	− 0.80 (− 0.99, − 0.62)
	S: MED	− 5.7 (− 8.1, − 3.4)	< .001	− 0.87 (− 1.21, − 0.54)
	S: PSY	− 0.8 (− 6.8, 5.2)	.779 ^NS^	− 0.13 (− 0.98, 0.77)
	S: MED + PSY	− 8.9 (− 15.1, − 2.7)	.007	− 1.34 (− 2.17, − 0.49)
	S: IBI-ONLY	− 5.3 (− 6.6, − 3.9)	< .001	− 0.80 (− 0.99, − 0.60)
GAD-7	OVERALL	− 2.4 (− 2.9, − 1.8)	< .001	− 0.62 (− 0.76, − 0.49)
	S: MED	− 2.6 (− 3.5, − 1.6)	< .001	− 0.67 (− 0.92, − 0.44)
	S: PSY	− 0.8 (− 3.2, 1.6)	.502 ^NS^	− 0.21 (− 0.81, 0.42)
	S: MED + PSY	− 4.9 (− 7.6, − 2.3)	< .001	− 1.29 (− 1.91, − 0.66)
	S: IBI-ONLY	− 2.3 (− 2.9, − 1.7)	< .001	− 0.60 (− 0.75, − 0.46)
IMET	OVERALL	− 8.1 (− 10.7, − 5.5)	< .001	− 0.53 (− 0.70, − 0.37)
	S: MED	− 10.3 (− 14.6, − 6.0)	< .001	− 0.68 (− 0.95, − 0.41)
	S: PSY	1.7 (− 8.5, 11.9)	.738 ^NS^	0.11 (− 0.51, 0.73)
	S: MED + PSY	− 20.6 (− 32.0, − 9.1)	.001	− 1.36 (− 2.11, − 0.61)
	S: IBI-ONLY	− 7.5 (− 10.2, − 4.8)	< .001	− 0.50 (− 0.67, − 0.33)
COSTA	OVERALL	− 5.8 (− 7.3, − 4.3)	< .001	− 0.57 (− 0.71, − 0.44)
	S: MED	− 7.7 (− 9.9, − 5.5)	< .001	− 0.75 (− 0.96, − 0.54)
	S: PSY	− 4.3 (− 10.6, 2.0)	.170 ^NS^	− 0.42 (− 1.03, 0.17)
	S: MED + PSY	− 14.0 (− 20.7, − 7.4)	< .001	− 1.37 (− 1.96, − 0.78)
	S: IBI-ONLY	− 5.1 (− 6.8, − 3.5)	< .001	− 0.50 (− 0.66, − 0.35)
PHQ-S	OVERALL	− 1.3 (− 1.8, − 0.8)	< .001	− 0.40 (− 0.54, − 0.24)
	S: MED	− 2.0 (− 2.9, − 1.1)	< .001	− 0.61 (− 0.87, − 0.36)
	S: PSY	− 0.9 (− 3.1, 1.4)	.443 ^NS^	− 0.26 (− 0.94, 0.41)
	S: MED + PSY	− 2.3 (− 4.8, 0.3)	.079 ^NS^	− 0.70 (− 1.43, 0.00)
	S: IBI-ONLY	− 1.1 (− 1.6, − 0.6)	< .001	− 0.34 (− 0.49, − 0.18)
GSE	OVERALL	2.0 (1.3, 2.7)	< .001	0.41 (0.27, 0.55)
	S: MED	2.5 (1.4, 3.7)	< .001	0.52 (0.29, 0.75)
	S: PSY	0.9 (− 2.1, 4.0)	.528 ^NS^	0.19 (− 0.36, 0.79)
	S: MED + PSY	3.7 (0.3, 7.1)	.035 ^NS^	0.76 (0.04, 1.41)
	S: IBI-ONLY	1.9 (1.2, 2.6)	< .001	0.38 (0.23, 0.52)
EURO-QOL	OVERALL	2.1 (1.5, 2.7)	< .001	0.51 (0.37, 0.66)
	S: MED	2.8 (1.6, 3.9)	< .001	0.68 (0.42, 0.96)
	S: PSY	0.2 (− 2.2, 2.6)	.894 ^NS^	0.04 (− 0.54, 0.58)
	S: MED + PSY	6.3 (3.2, 9.3)	< .001	1.53 (0.75, 2.24)
	S: IBI-ONLY	1.9 (1.2, 2.5)	< .001	0.46 (0.30, 0.61)
BSSS-s	OVERALL	1.3 (0.8, 1.7)	< .001	0.35 (0.22, 0.47)
	S: MED	1.5 (0.8, 2.3)	< .001	0.42 (0.22, 0.62)
	S: PSY	− 0.5 (− 2.4, 1.5)	.638 ^NS^	− 0.13 (− 0.65, 0.38)
	S: MED + PSY	2.3 (0.2, 4.3)	.033 ^NS^	0.63 (0.06, 1.21)
	S: IBI-ONLY	1.2 (0.8, 1.7)	< .001	0.34 (0.21, 0.47)
BSSS-e	OVERALL	1.0 (0.3, 1.6)	.003	0.18 (0.07, 0.29)
	S: MED	0.8 (− 0.2, 1.8)	.104 ^NS^	0.15 (− 0.03, 0.32)
	S: PSY	1.0 (− 1.4, 3.4)	.389 ^NS^	0.19 (− 0.25, 0.60)
	S: MED + PSY	0.9 (− 1.4, 3.2)	.443 ^NS^	0.16 (− 0.26, 0.57)
	S: IBI-ONLY	1.0 (0.3, 1.8)	.006 ^NS^	0.19 (0.06, 0.32)

Between-arm effects are presented for all randomized individuals (OVERALL) and each stratum: individuals taking medication (S: MED), receiving psychotherapy (S: PSY), receiving both psychotherapy and medication (S: MED + PSY), and those not receiving either psychotherapy or medication (S: IBI-ONLY). Positive between-arm differences indicate “in favor of the INT-arm” for GSE, EUROHIS-QOL, and BSSS. For all other outcomes, negative between-arm differences indicate “in favor of the INT-arm.” ∆ = unstandardized between-arm difference. *d* = Cohen’s d-like effect measure calculated by dividing ∆ by the baseline standard deviation

*95% CI* 95% confidence interval, *BDI-II* Beck-Depression-Inventory-II, *BSSS-e* Berlin Social Support Scale – emotional support, *BSSS-s* Berlin Social Support Scale – support-seeking behavior, *COSTA* Cognitive Style Assessment, *EUROHIS-QOL* European Health Interview Survey Quality of Life 8-item index, *GAD-7* Generalized Anxiety Scale, *GSE* General Self-Efficacy, *IMET* Index for Measurement of Impairments in Participation, *PHQ-S* PHQ Stress Module, *PHQ-9* Patient Health-Questionnaire 9, ^*NS*^ non-significant after Bonferroni correction

**Table 6:** Rates of meaningful improvements and deterioration

	PHQ-9			BDI-II		
	*n* (%) CAU	*n* (%) INT	ARD in %-points (95% CI)	*n* (%) CAU	*n* (%) INT	ARD in %-points (95% CI)
Improvements						
OVERALL	132.4 (14.3%)	375.8 (38.5%)	24.2 (20.1 to 28.2)	137.2 (14.8%)	435.2 (44.6%)	29.8 (23.8 to 35.7%)
S: MED	25.4 (14.3%)	75 (39.5%)	25.1 (15.5 to 34.2)	26.8 (15.1%)	90.7 (47.7%)	32.6 (21.7 to 42.8%)
S: PSY	6.5 (15.4%)	10.7 (34.6%)	19.2 (− 3.0 to 42.2)	6.4 (15.1%)	11.5 (37.0%)	21.8 (− 2.9 to 49.0%)
S: MED + PSY	2.4 (9.4%)	14.3 (54.8%)	45.4 (15.2 to 67.1)	4.8 (19%)	18.6 (71.5%)	52.5 (17.7 to 73.3%)
S: IBI-ONLY	98.2 (14.4%)	275.9 (37.9%)	23.5 (18.7 to 28.1)	99.3 (14.6%)	314.4 (43.2%)	28.6 (22.1 to 35.1%)
Deterioration						
OVERALL	46.8 (5.1%)	40 (4.1%)	− 1.0 (− 3.1 to 1.2)	19 (2.1%)	33.3 (3.4%)	1.4 (− 0.5 to 3.8)
S: MED	7.7 (4.3%)	7.4 (3.9%)	− 0.4 (− 5.3 to 4.8)	2.5 (1.4%)	9.4 (4.9%)	3.6 (− 0.8 to 9.3)
S: PSY	1.1 (2.5%)	1.5 (4.8%)	2.2 (− 9.3 to 25.4)	0.1 (0.2%)	1.7 (5.6%)	5.4 (− 12.2 to 28.7)
S: MED + PSY	1.2 (4.6%)	0.3 (1.0%)	− 3.6 (− 22.4 to 25.1)	0 (0.2%)	0.5 (1.8%)	1.6 (− 26.2 to 29.5)
S: IBI-ONLY	36.9 (5.4%)	30.8 (4.2%)	− 1.2 (− 3.7 to 1.3)	16.4 (2.4%)	21.7 (3.0%)	0.6 (− 1.5 to 3.2)

Rates and absolute risk differences in meaningful improvements (at least 50% symptom improvement) and deterioration (5 PHQ-9 or 10 BDI-II points deterioration) are presented for all randomized individuals (OVERALL) and each stratum: individuals taking medication (S: MED), receiving psychotherapy (S: PSY), receiving both psychotherapy and medication (S: MED + PSY), and those not receiving either psychotherapy or medication (S: IBI-ONLY). Note that frequencies are mean frequencies over all imputed datasets. Thus, counts have digits

*BDI-II* Beck-Depression-Inventory-II, *CAU* unstandardized Care-as-Usual control condition, *INT* Intervention condition, *PHQ-9* Patient-Health-Questionnaire 9

#### Symptoms of depression—all randomized individuals

Symptoms of depression, measured with the PHQ-9 and BDI-II, decreased in the INT and CAU-arm within 11 weeks after randomization (see Table 4). In the INT-arm, the average PHQ-9 score decreased from *moderate* to *mild* depressive symptoms (*M*_*T0*_ = 12.8, *M*_*WEEK11*_ = 8.3, ∆_*WEEK11*-T0_ =  − 4.5, *p* < 0.001), while it remained *moderate* in the CAU-arm (*M*_*T0*_ = 13.0, *M*_*WEEK11*_ = 10.9, ∆_*WEEK11*-T0_ =  − 2.1, *p* < 0.001). Similarly, the average BDI-II score decreased from *moderate* to *mild* depressive symptoms in the INT-arm (*M*_*T0*_ = 25.1, *M*_*WEEK11*_ = 15.0, ∆_*WEEK11*-T0_ =  − 10.1, *p* < 0.001) and remained *moderate* in the CAU-arm (*M*_*T0*_ = 25.2, *M*_*WEEK11*_ = 20.4, ∆_*WEEK11*-T0_ =  − 4.8, *p* < 0.001).

The baseline-adjusted between-arm differences at 11 weeks after randomization (i.e., the relative effect of providing individuals access to the IBI: ∆_BET_; see Table 5) were estimated at ∆_BET_ =  − 2.5 PHQ-9 points, 95% CI (− 2.9, − 2.0), *p* < 0.001, *d* =  − 0.7, and ∆_BET_ =  − 5.3 BDI-II points, 95% CI (− 6.5, − 4.1), *p* < 0.001, *d* =  − 0.8. Thus, these results favor the INT over the CAU-arm.

Moreover, individuals in the INT-arm had a higher probability of experiencing meaningful symptom improvements (i.e., at least 50% symptom reduction compared to baseline): PHQ-9: 14.3% (CAU) vs. 38.5% (INT), ARD = 24.2%-points; BDI-II: 14.8% (CAU) vs. 44.6% (INT), ARD = 29.8%-points. Again, the results favor the INT over the CAU-arm (see Table 6).

In the sensitivity analysis, when the imputed values in the INT-arm were increased by 50%, the BDI-II between-arm effect decreased to ∆_BET_ =  − 4.1, 95% CI (− 5.5, − 2.7), *p* < 0.001, *d* =  − 0.6, and the PHQ-9 between-arm effect to ∆_BET_ =  − 1.8, 95% CI (− 2.2, − 1.3), *p* < 0.001, *d* =  − 0.5. The rates of meaningful improvements in the INT-arm also decreased to 35.3% (PHQ-9, ARD = 20.9%-points) and 41.9% (BDI-II, ARD = 27.0%-points). However, the results still favor the INT-arm (see supplemental Tables).

Overall, the rates of suicidal ideations and symptom deterioration were low. 24.1% (235 of 975) of the individuals in the INT-arm reported mild suicidal ideation at least once (PHQ-9 item 9 = 1), and 2.3% (22 of 975) reported more severe suicidal ideation at least once (PHQ-9 item 9 ≥ 2). The deterioration (i.e., increases of 5 PHQ-9/10 BDI-II points) rates in the INT-arm were 3.4% (BDI-II) and 4.1% (PHQ-9; see Table 6 for all rates).

#### Symptoms of depression – stratified effect estimates

The INT-arm showed larger average within-arm changes than the CAU-arm in all strata: PHQ-9: MED: − 4.9 vs. − 2.3 points; PSY: − 3.7 vs. − 3.0 points; PSY + MED: − 6.1 vs. − 2.6 points; IBI-ONLY: − 4.4 vs. − 2.0 points; BDI-II: MED: − 11.1 vs. − 5.0; PSY =  − 8.0 vs. − 6.2; MED + PSY: − 14.1 vs. − 5.9; IBI-ONLY =  − 9.8 vs. − 4.7 points (see Table 4).

The adjusted between-arm differences were largest for the MED + PSY stratum, ∆_BET_ =  − 3.8 PHQ-9 points, 95% CI (− 6.6, − 1.1), *p* = 0.008, *d* =  − 1.1, and ∆_BET_ =  − 8.9 BDI-II points, 95% CI (− 15.1, − 2.7), *p* = 0.007, *d* =  − 1.3, and smallest for the PSY stratum ∆_BET_ =  − 0.8 PHQ-9 points, 95% CI (− 3.1, 1.5), *p* = 0.478, *d* =  − 0.2, and ∆_BET_ =  − 0.8 BDI-II points, 95% CI (− 6.8, 5.2), *p* = 0.779, *d* =  − 0.1. The effects for the other strata were in between and similar to those observed in the entire sample. After Bonferroni correction, all but the between-arm effects for the PSY stratum (BDI-II and PHQ: *p* > 0.025) were statistically significant.

Similarly, the rates of meaningful improvements were smallest in the PSY stratum (BDI-II: 37.0%, PHQ-9: 34.6%) and largest in the MED + PSY stratum (BDI-II: 71.5%, PHQ-9: 54.8%). The between-arm differences in rates of meaningful improvements in the PHQ-9 were 45.4%-points (MED + PSY), 25.1%-points (MED), 23.5%-points (IBI-ONLY), and 19.2%-points (PSY). The same order emerged for the BDI-II: 52.5%-points (MED + PSY), 32.6%-points (MED), 28.6%-points (IBI-ONLY), and 21.8%-points (PSY). All ARDs, except those for the PSY stratum, are statistically significant (i.e., the confidence interval does not include zero), favoring the INT-arm.

The sensitivity analysis revealed that increasing the imputed BDI-II and PHQ-9 scores in the MED and IBI-ONLY strata did not turn the between-arm differences non-significant, albeit effects decreased (see supplemental material). The effects in the MED + PSY stratum turned non-significant after increasing the imputed PHQ-9 scores by 30% and the imputed BDI-II scores by 40%. This finding indicates that our conclusions are robust against large deviations from the MAR assumption implied throughout the imputation process.

### Secondary outcome measures

Considering all randomized individuals, the secondary outcomes showed larger within-arm improvements in the INT than in the CAU-arm (see Table 4). The absolute values of the standardized within-arm effect estimates ranged between *d* = 0.3 (social support) to 1.1 (anxiety; median *d* = 0.7) in the INT-arm and from *d* = 0.1 (social support) to 0.5 (anxiety; median *d* = 0.2) in the CAU-arm. The absolute values of the standardized between-arm effect estimates ranged between *d* = 0.2 (social support) and 0.6 (anxiety), favoring the INT-arm (all *p* < 0.003).

A similar pattern emerged across most strata. However, after applying Bonferroni correction, many effects in the PSY and MED + PSY strata were non-significant (*p* > 0.006; see Table 5). However, the point estimates point towards a benefit of the INT over the CAU-arm.

### Acceptability and self-reported side-effects

A large majority (80%) of the participants assigned to the INT-arm were satisfied with the IBI (completely satisfied: 8.9%; very satisfied: 33.7%; satisfied: 37.0%; less satisfied 17.8%; not satisfied: 2.6%), and the majority would recommend the program to other individuals with depression (*definitely*: 42.0%; *very likely*: 29.0%; likely: 17.0%; *likely not* 9.9%; *definitely not*: 2.1%). Moreover, 21.3% rated the IBI as *very helpful*, 31.1% as helpful, 23.8% as *mostly helpful,* 16.6% *as mostly not helpful,* and 7.2% as *not helpful*. Overall, 10.5% of participants reported negative side effects. The consequences of these side effects for their well-being at the time of occurrence were rated as *non-existent* (4.8%), *slightly negative* (10.5%), *negative* (52.3%), or *very negative* (32.4%). Similarly, the consequences for their current well-being (at the point of assessment post-intervention) were rated as *non-existent* (44.6%), *slightly negative* (20.0%), *negative* (27.8%), or *very negative* (7.7%).

## Discussion

The study investigated the effectiveness of an IBI for adults (≥ 18 years) with mild (23%), moderate (47%), and severe (30%) self-reported symptoms of depression as measured with the BDI-II. All participants actively sought support, considering the IBI a viable treatment. The reported effects are informative from a treatment policy perspective by describing the expected health benefits of providing access to an IBI for individuals with mild to moderately severe depression (without psychosis, mania, or acute suicidal ideations) 9 to 11 weeks after randomization, regardless of adherence and usage of other parallel treatments.

### Symptoms of depression

Considering the entire sample, the IBI yielded larger average symptom improvements (differences to CAU: − 5.3 BDI-II points; − 2.5 PHQ-9 points) and higher rates of meaningful improvements (PHQ-9: 38.5% vs 14.3%, BDI-II 44.6% vs. 14.8%) compared to CAU. Therefore, our results align with a recent meta-analysis [6], which showed that guided IBI outperforms waitlists by 3.3 PHQ-9 points and CAU by 1.7 PHQ-9 points, with response rates of 52% for guided IBI, 18% for waitlists, and 26% for CAU. Note that our model made conservative assumptions in the imputation process. Note that our estimates of the main outcome are best interpreted as the lower bound of benefits that result from providing individuals access to an IBI, as they consider imperfect adherence. Our sensitivity analysis confirmed the robustness of the interventions’ benefit over CAU. Even after increasing the imputed values by 50%, the results favored the INT- over the CAU-arm.

Our analysis provides stratified effect estimates for individuals using the IBI as a stand-alone or add-on intervention. The results are informative as to whether individuals currently receiving another depression treatment should get access to an IBI. Individuals using the IBI as an add-on to antidepressants showed benefits of − 2.7 PHQ-9 and − 5.7 BDI-II points when compared to individuals using antidepressants only. This finding is consistent with Mantani et al. [12], who reported around 2 PHQ-9 points benefit from adding IBI to individuals switching their antidepressants. While our results also align with Klein et al. [10], who reported a benefit of 1.2 PHQ-9 points, they differ from those of Meyer et al. [13], who reported about 5 PHQ-9 points benefits when adding IBI to medication. A possible explanation is that Meyer et al. included more severely impaired individuals. Moreover, we found that adding the IBI to antidepressants mono-treatment leads to improvements in secondary outcomes such as self-reported quality of life. This finding aligns with research indicating that adding psychological treatments addresses other problem areas than antidepressants [17, 18]

The results are inconclusive regarding IBI as an add-on to PSY or PSY + MED. While the add-on effects in the PSY strata are close to zero (− 0.8 BDI-II and PHQ-9 points), those in the PSY + MED stratum are larger (− 3.8 PHQ-9 and − 8.9 BDI-II points) and comparable to those reported by Berger et al. [30]. Potential explanations include the small strata sizes (e.g., the PSY and PSY + MED sample are only large enough to depict large between-arm effects with sufficient power), which may lead to imprecise estimates, differential adherence patterns (individuals in the PSY stratum completed fewer modules in the 11 weeks after randomization than individuals in the MED + PSY strata), and the missing random assignment of the secondary treatments. Thus, the underlying populations might differ. Future studies must assign the treatment packages randomly to address these limitations. Nevertheless, although all strata-specific effect estimates should be interpreted carefully, they suggest no reason to exclude individuals who enter an IBI with a concurrent treatment.

### Secondary outcome measures

Concerning the three large strata (i.e., the overall sample, the medication-only, and the IBI-only strata), a fairly consistent pattern emerged concerning the secondary outcome measures. Relative treatment effects were largest on measures focusing on pathology and impairments (i.e., anxiety, restrictions in participation, cognitive biases; mostly *d* > 0.50), somewhat smaller in self-reported quality of life, self-efficacy, and stress (mostly 0.30 < *d* < 0.50). Furthermore, the effects in self-reported emotional social support were smallest across strata and failed to reach statistical significance in the MED stratum. Overall, the pattern is intuitive, given the intervention’s focus on reducing depression and its associated impairments. However, consistent with findings from other studies, our results suggest that IBIs have beneficial effects on secondary outcomes. The non-significant effect in social support could be explained by the fact that participants already reported high baseline values.

The point estimates in the smaller strata (MED + PSY, PSY) mainly favor the IBI over CAU. However, most estimates fail to reach statistical significance. Again, the same limitations discussed in the context of symptoms of depression should be considered. Therefore, one may interpret these findings as promising, although investigations in larger samples are needed. These studies should consider the improvements in study design discussed above.

### Adherence

Regarding adherence, 62.5% of all randomized subjects accessed all treatment modules within 11 weeks after randomization, which increased to 77.5% when ignoring the time needed to complete the modules. These rates align with previous studies using the same intervention (~ 76 to 83%, [8, 9]) and meta-analytically findings for guided IBIs (~ 75%, [6]). These findings suggest that, on average, participants need longer than the initially intended eight weeks. At the same time, our results indicate that, on average, improvements in depressive symptoms can be observed, although not all participants complete the program as intended and work at their own pace. However, this finding does not prove that individuals should get more time to work with the program. In fact, our study does not provide data to conclude on the dose–response relationship, as the dose (i.e., how participants engaged with the IBI) is self-selected and not randomly assigned. Therefore, studies inspecting the dose–response relationship in IBIs are needed. For example, a study could compare the 3-month depressive symptom load between two study arms that either use IBI-based single-session interventions or multiple-module IBI in which participant work at their own pace. Such studies would provide insights into which IBI format is the most effective.

The stratified analysis showed lower completion rates within 11 weeks after randomization for those receiving the IBI as an add-on to psychotherapy (PSY: 41.9%; MED + PSY: 53.8%), although overall completion rates (i.e., when ignoring the time needed to complete the modules) were comparable to those of the other strata (> 70%). Thus, individuals using IBI as an add-on to PSY require more time to complete the treatment. This delay in completion could be due to the demands that two concurrent and non-integrated psychotherapeutic treatments impose on participants’ time and emotional resources.

Overall, participants were satisfied with the program, would recommend it to other depressed individuals with depression, and rated the program as helpful. However, ~ 11 in 100 participants report negative side effects. This number is comparable to the 8.6% reported in a previous study using the same intervention in less impaired individuals [31].

### Limitations

The trial has the following limitations. First, all individuals are self-selected, limiting generalizability to those considering IBI as a viable treatment alternative. Given the impact of treatment preferences (e.g., [32]), future trials should include preference-based randomization. Second, the interpretation of strata-specific effects (secondary analysis) should consider that (a) add-on treatments were not randomly assigned but represented the treatment status at randomization, and (b) the analysis did not consider whether the treatment might have changed throughout the intervention. However, our results do not show which treatment combinations are most beneficial. Further trials randomizing treatment combinations are warranted. Third, the current trial excluded individuals with a history of bipolar disorder and psychosis and applied restrictive inclusion criteria on suicidal ideations. Thus, our conclusions are limited to a specific subgroup of depressed individuals and not to individuals with depression in general. Fourth, our analysis precludes conclusions on controlled long-term effects (e.g., compared to individuals with only access to CAU within a 12-month treatment period). However, other research indicates that IBIs yield sustainable symptom improvements [6]. Fifth, individuals could access the intervention after the waiting period; thus, the immediate relief may confound the estimates [32] and influence the treatment-seeking behavior (e.g., no active search for treatment alternatives) while waiting.

## Conclusion

In conclusion, our results align with other studies showing that providing interested adults access to therapist-guided, cognitive-behavioral IBIs leads to beneficial health outcomes, no matter if individuals use the IBI as a stand-alone or add-on intervention to another evidence-based treatment. Therefore, IBI should be considered a valid and low-threshold treatment option.

## Supplementary Information

Supplementary Material 1.

## Data Availability

The data is available on reasonable request. Please contact MH (manuel.heinrich[at]fu-berlin.de).

## Abbreviations

ARDs: Absolute risk differences
BDI-II: Beck-Depression-Inventory II
BSSS: Berlin Social Support-Scale
CAU: Care-as-Usual
COSTA: Cognitive Styles Assessment
IBI: Internet-based Intervention
INT: Intervention Arm
GSE: Generalized Self-Efficacy Scale
IMET: Index for Measurement of Impairments in Participation
MDE: Major Depressive Episode
MED: Antidepressant Medication
PHQ-9: Patient-Health-Questionnaire 9
PSY: Psychotherapy
PSY + MED: Psychotherapy + Antidepressant Medication
RCT: Randomized-Controlled Trial
