# Supplementary material for: A randomized controlled trial of a therapist-guided online intervention for depressed adults and its utility as an adjunctive to antidepressants and psychotherapy
Source: BMC Psychiatry. 2025 Feb 11;25:116. doi: 10.1186/s12888-025-06564-2 (PMC11817706; doi:10.1186/s12888-025-06564-2)
Supplement: Supplementary file 1 — Supplementary Material 1. [file 12888_2025_6564_MOESM1_ESM.docx]

| **Supplemental Table S1.** Results of the sensitivity analysis for the PHQ-9. | | | | |
| --- | --- | --- | --- | --- |
| Condition | Strata | ∆ (95% CI) | p | d (95% CI) |
| PHQ-9 (MAR) | OVERALL | −2.5 (−2.9, −2.0) | <.001 | −0.70 (−0.82, −0.58) |
| + 10% | OVERALL | −2.3 (−2.7, −1.9) | <.001 | −0.66 (−0.78, −0.54) |
| + 20% | OVERALL | −2.2 (−2.6, −1.8) | <.001 | −0.62 (−0.74, −0.50) |
| + 30% | OVERALL | −2.0 (−2.5, −1.6) | <.001 | −0.58 (−0.71, −0.46) |
| + 40% | OVERALL | −1.9 (−2.4, −1.5) | <.001 | −0.55 (−0.67, −0.42) |
| + 50% | OVERALL | −1.8 (−2.2, −1.3) | <.001 | −0.51 (−0.64, −0.38) |
| PHQ-9 (MAR) | MED | −2.7 (−3.7, −1.7) | <.001 | −0.77 (−1.04, −0.50) |
| + 10% | MED | −2.6 (−3.6, −1.6) | <.001 | −0.73 (−1.00, −0.45) |
| + 20% | MED | −2.4 (−3.4, −1.4) | <.001 | −0.69 (−0.97, −0.41) |
| + 30% | MED | −2.3 (−3.3, −1.2) | <.001 | −0.65 (−0.94, −0.36) |
| + 40% | MED | −2.2 (−3.2, −1.1) | <.001 | −0.62 (−0.90, −0.32) |
| + 50% | MED | −2.0 (−3.1, −0.9) | <.001 | −0.58 (−0.87, −0.28) |
| PHQ-9 (MAR) | PSY | −0.8 (−3.1, 1.5) | .478 | −0.23 (−0.89, 0.40) |
| + 10% | PSY | −0.6 (−3.0, 1.7) | .589 | −0.18 (−0.85, 0.48) |
| + 20% | PSY | −0.5 (−2.9, 2.0) | .707 | −0.13 (−0.81, 0.55) |
| + 30% | PSY | −0.3 (−2.8, 2.2) | .825 | −0.08 (−0.77, 0.62) |
| + 40% | PSY | −0.1 (−2.7, 2.5) | .937 | −0.03 (−0.74, 0.69) |
| + 50% | PSY | 0.1 (−2.6, 2.7) | .955 | 0.02 (−0.71, 0.77) |
| PHQ-9 (MAR) | MED+PSY | −3.8 (−6.6, −1.1) | .008 | −1.09 (−1.78, −0.43) |
| + 10% | MED+PSY | −3.6 (−6.5, −0.8) | .014 | −1.04 (−1.74, −0.36) |
| + 20% | MED+PSY | −3.4 (−6.4, −0.5) | .023 | −0.98 (−1.69, −0.27) |
| + 30% | MED+PSY | −3.3 (−6.3, −0.2) | .036 | −0.93 (−1.65, −0.19) |
| + 40% | MED+PSY | −3.1 (−6.2, 0.0) | .053 | −0.88 (−1.61, −0.11) |
| + 50% | MED+PSY | −2.9 (−6.1, 0.3) | .075 | −0.82 (−1.57, −0.03) |
| PHQ-9 (MAR) | IBI-ONLY | −2.4 (−2.9, −2.0) | <.001 | −0.69 (−0.82, −0.56) |
| + 10% | IBI-ONLY | −2.3 (−2.8, −1.8) | <.001 | −0.65 (−0.78, −0.52) |
| + 20% | IBI-ONLY | −2.2 (−2.6, −1.7) | <.001 | −0.62 (−0.75, −0.48) |
| + 30% | IBI-ONLY | −2.0 (−2.5, −1.5) | <.001 | −0.58 (−0.71, −0.44) |
| + 40% | IBI-ONLY | −1.9 (−2.4, −1.4) | <.001 | −0.55 (−0.68, −0.40) |
| + 50% | IBI-ONLY | −1.8 (−2.3, −1.3) | <.001 | −0.51 (−0.65, −0.37) |
| **Note.** Results of the sensitivity analysis. +10% indicates that the scores imputed using multiple imputation for individuals in the INT arm—excluding those already imputed alongside the CAU group—were increased by 10%. Similarly, +20% indicates that the imputed scores used in the primary analysis reported in the manuscript were increased by 20%. This approach simulates a Not Missing at Random (NMAR) scenario, where the imputations may underestimate the symptom burden of individuals in the INT-arm, potentially leading to an overestimation of the between-group effect. The analysis explores the tipping point at which the conclusions change. | | | | |

| **Supplemental Table S2.** Results of the sensitivity analysis for the BDI-II. | | | | |
| --- | --- | --- | --- | --- |
| Condition | Strata | ∆ (95% CI) | p | d (95% CI) |
| BDI-II (MAR) | OVERALL | −5.3 (−6.5, −4.1) | <.001 | −0.80 (−0.99, −0.62) |
| + 10% | OVERALL | −5.1 (−6.3, −3.8) | <.001 | −0.77 (−0.96, −0.58) |
| + 20% | OVERALL | −4.8 (−6.1, −3.5) | <.001 | −0.73 (−0.93, −0.54) |
| + 30% | OVERALL | −4.6 (−5.9, −3.2) | <.001 | −0.69 (−0.90, −0.50) |
| + 40% | OVERALL | −4.3 (−5.7, −3.0) | <.001 | −0.66 (−0.86, −0.46) |
| + 50% | OVERALL | −4.1 (−5.5, −2.7) | <.001 | −0.62 (−0.83, −0.42) |
| BDI-II (MAR) | MED | −5.7 (−8.1, −3.4) | <.001 | −0.87 (−1.21, −0.54) |
| + 10% | MED | −5.5 (−7.9, −3.1) | <.001 | −0.83 (−1.18, −0.50) |
| + 20% | MED | −5.2 (−7.7, −2.8) | <.001 | −0.79 (−1.14, −0.45) |
| + 30% | MED | −5.0 (−7.5, −2.5) | <.001 | −0.75 (−1.11, −0.40) |
| + 40% | MED | −4.7 (−7.3, −2.2) | <.001 | −0.71 (−1.08, −0.35) |
| + 50% | MED | −4.5 (−7.1, −1.9) | <.001 | −0.68 (−1.05, −0.30) |
| BDI-II (MAR) | PSY | −0.8 (−6.8, 5.2) | .779 | −0.13 (−0.98, 0.77) |
| + 10% | PSY | −0.5 (−6.6, 5.7) | .880 | −0.07 (−0.95, 0.85) |
| + 20% | PSY | −0.1 (−6.5, 6.3) | .978 | −0.01 (−0.91, 0.93) |
| + 30% | PSY | 0.3 (−6.3, 6.9) | .930 | 0.04 (−0.88, 1.02) |
| + 40% | PSY | 0.7 (−6.2, 7.5) | .846 | 0.10 (−0.86, 1.11) |
| + 50% | PSY | 1.0 (−6.0, 8.1) | .769 | 0.15 (−0.83, 1.20) |
| BDI-II (MAR) | MED+PSY | −8.9 (−15.1, −2.7) | .007 | −1.34 (−2.17, −0.49) |
| + 10% | MED+PSY | −8.5 (−14.9, −2.2) | .011 | −1.30 (−2.13, −0.42) |
| + 20% | MED+PSY | −8.2 (−14.8, −1.6) | .016 | −1.25 (−2.10, −0.35) |
| + 30% | MED+PSY | −7.9 (−14.7, −1.1) | .024 | −1.20 (−2.07, −0.28) |
| + 40% | MED+PSY | −7.6 (−14.6, −0.6) | .035 | −1.15 (−2.05, −0.19) |
| + 50% | MED+PSY | −7.3 (−14.5, −0.0) | .049 | −1.10 (−2.02, −0.11) |
| BDI-II (MAR) | IBI-ONLY | −5.3 (−6.6, −3.9) | <.001 | −0.80 (−0.99, −0.60) |
| + 10% | IBI-ONLY | −5.0 (−6.4, −3.7) | <.001 | −0.76 (−0.96, −0.56) |
| + 20% | IBI-ONLY | −4.8 (−6.2, −3.4) | <.001 | −0.73 (−0.93, −0.52) |
| + 30% | IBI-ONLY | −4.6 (−6.0, −3.1) | <.001 | −0.69 (−0.90, −0.48) |
| + 40% | IBI-ONLY | −4.3 (−5.8, −2.9) | <.001 | −0.65 (−0.87, −0.44) |
| + 50% | IBI-ONLY | −4.1 (−5.6, −2.6) | <.001 | −0.62 (−0.84, −0.40) |
| **Note.** Results of the sensitivity analysis. +10% indicates that the scores imputed using multiple imputation for individuals in the INT arm—excluding those already imputed alongside the CAU group—were increased by 10%. Similarly, +20% indicates that the imputed scores used in the primary analysis reported in the manuscript were increased by 20%. This approach simulates a Not Missing at Random (NMAR) scenario, where the imputations may underestimate the symptom burden of individuals in the INT-arm, potentially leading to an overestimation of the between-group effect. The analysis explores the tipping point at which the conclusions change. | | | | |

| **Supplemental Table S3.** Results of the sensitivity analysis for the PHQ-9 (response rates). | | | | |
| --- | --- | --- | --- | --- |
| Group | Scale | CON, n (%) | INT, n (%) | Δ, 95% CI |
| PHQ-9 (MAR) | OVERALL | 132.4 (14.3%) | 375.8 (38.5%) | 24.2 (20.1 to 28.2) |
| + 10% | OVERALL | 132.4 (14.3%) | 363.9 (37.3%) | 23.0 (18.9 to 27.0) |
| + 20% | OVERALL | 132.4 (14.3%) | 356.5 (36.6%) | 22.2 (18.1 to 26.2) |
| + 30% | OVERALL | 132.4 (14.3%) | 352.1 (36.1%) | 21.8 (17.7 to 25.8) |
| + 40% | OVERALL | 132.4 (14.3%) | 345.4 (35.4%) | 21.1 (17.0 to 25.1) |
| + 50% | OVERALL | 132.4 (14.3%) | 343.7 (35.3%) | 20.9 (16.8 to 24.9) |
| PHQ-9 (MAR) | MED | 25.4 (14.3%) | 75 (39.5%) | 25.1 (15.5 to 34.2) |
| + 10% | MED | 25.4 (14.3%) | 72.8 (38.3%) | 24.0 (14.4 to 33.0) |
| + 20% | MED | 25.4 (14.3%) | 70.9 (37.3%) | 23.0 (13.5 to 32.0) |
| + 30% | MED | 25.4 (14.3%) | 69.6 (36.7%) | 22.3 (12.8 to 31.4) |
| + 40% | MED | 25.4 (14.3%) | 68.5 (36.1%) | 21.7 (12.2 to 30.8) |
| + 50% | MED | 25.4 (14.3%) | 68.3 (36%) | 21.6 (12.1 to 30.7) |
| PHQ-9 (MAR) | PSY | 6.5 (15.4%) | 10.7 (34.6%) | 19.2 (−3.0 to 42.2) |
| + 10% | PSY | 6.5 (15.4%) | 10.1 (32.6%) | 17.2 (−4.6 to 40.3) |
| + 20% | PSY | 6.5 (15.4%) | 10 (32.2%) | 16.8 (−5.0 to 40.0) |
| + 30% | PSY | 6.5 (15.4%) | 9.9 (31.9%) | 16.5 (−5.2 to 39.7) |
| + 40% | PSY | 6.5 (15.4%) | 9.7 (31.3%) | 15.9 (−5.5 to 38.5) |
| + 50% | PSY | 6.5 (15.4%) | 9.6 (30.9%) | 15.5 (−5.7 to 37.8) |
| PHQ-9 (MAR) | MED+PSY | 2.4 (9.4%) | 14.3 (54.8%) | 45.4 (15.2 to 67.1) |
| + 10% | MED+PSY | 2.4 (9.4%) | 13.5 (51.9%) | 42.5 (12.7 to 64.7) |
| + 20% | MED+PSY | 2.4 (9.4%) | 13.2 (50.8%) | 41.3 (11.7 to 63.8) |
| + 30% | MED+PSY | 2.4 (9.4%) | 13.1 (50.2%) | 40.8 (11.2 to 63.3) |
| + 40% | MED+PSY | 2.4 (9.4%) | 12.6 (48.5%) | 39.0 (9.8 to 61.8) |
| + 50% | MED+PSY | 2.4 (9.4%) | 12.5 (47.9%) | 38.5 (9.5 to 61.0) |
| PHQ-9 (MAR) | IBI-ONLY | 98.2 (14.4%) | 275.9 (37.9%) | 23.5 (18.7 to 28.1) |
| + 10% | IBI-ONLY | 98.2 (14.4%) | 267.6 (36.8%) | 22.3 (17.6 to 26.9) |
| + 20% | IBI-ONLY | 98.2 (14.4%) | 262.4 (36%) | 21.6 (16.9 to 26.2) |
| + 30% | IBI-ONLY | 98.2 (14.4%) | 259.5 (35.6%) | 21.2 (16.5 to 25.8) |
| + 40% | IBI-ONLY | 98.2 (14.4%) | 254.6 (35%) | 20.5 (15.8 to 25.1) |
| + 50% | IBI-ONLY | 98.2 (14.4%) | 253.3 (34.8%) | 20.4 (15.7 to 25.0) |
| **Note.** Results of the sensitivity analysis. +10% indicates that the scores imputed using multiple imputation for individuals in the INT arm—excluding those already imputed alongside the CAU group—were increased by 10%. Similarly, +20% indicates that the imputed scores used in the primary analysis reported in the manuscript were increased by 20%. This approach simulates a Not Missing at Random (NMAR) scenario, where the imputations may underestimate the symptom burden of individuals in the INT-arm, potentially leading to an overestimation of the between-group effect. The analysis explores the tipping point at which the conclusions change. | | | | |

| **Supplemental Table S4.** Results of the sensitivity analysis for the BDI-II (response rates). | | | | |
| --- | --- | --- | --- | --- |
| Group | Scale | CON, n (%) | INT, n (%) | Δ, 95% CI |
| BDI-II (MAR) | OVERALL | 137.2 (14.8%) | 435.2 (44.6%) | 29.8 (23.8 to 35.7) |
| + 10% | OVERALL | 137.2 (14.8%) | 427.2 (43.8%) | 29.0 (23.1 to 34.9) |
| + 20% | OVERALL | 137.2 (14.8%) | 420.6 (43.1%) | 28.3 (22.5 to 34.1) |
| + 30% | OVERALL | 137.2 (14.8%) | 415.8 (42.6%) | 27.8 (22.1 to 33.6) |
| + 40% | OVERALL | 137.2 (14.8%) | 411.8 (42.2%) | 27.4 (21.7 to 33.1) |
| + 50% | OVERALL | 137.2 (14.8%) | 408.4 (41.9%) | 27.0 (21.4 to 32.7) |
| BDI-II (MAR) | MED | 26.8 (15.1%) | 90.7 (47.7%) | 32.6 (21.7 to 42.8) |
| + 10% | MED | 26.8 (15.1%) | 88.8 (46.7%) | 31.6 (20.9 to 41.6) |
| + 20% | MED | 26.8 (15.1%) | 87.7 (46.2%) | 31.0 (20.4 to 41.1) |
| + 30% | MED | 26.8 (15.1%) | 86.8 (45.7%) | 30.5 (19.9 to 40.6) |
| + 40% | MED | 26.8 (15.1%) | 86.1 (45.3%) | 30.1 (19.5 to 40.2) |
| + 50% | MED | 26.8 (15.1%) | 85.5 (45%) | 29.9 (19.3 to 39.9) |
| BDI-II (MAR) | PSY | 6.4 (15.1%) | 11.5 (37%) | 21.8 (−2.9 to 49.0) |
| + 10% | PSY | 6.4 (15.1%) | 11.1 (35.7%) | 20.6 (−3.5 to 47.3) |
| + 20% | PSY | 6.4 (15.1%) | 10.9 (35.1%) | 20.0 (−3.9 to 46.4) |
| + 30% | PSY | 6.4 (15.1%) | 10.6 (34.1%) | 19.0 (−4.5 to 45.3) |
| + 40% | PSY | 6.4 (15.1%) | 10.5 (33.8%) | 18.7 (−4.7 to 45.0) |
| + 50% | PSY | 6.4 (15.1%) | 10.4 (33.5%) | 18.4 (−4.9 to 44.6) |
| BDI-II (MAR) | MED+PSY | 4.8 (19%) | 18.6 (71.5%) | 52.5 (17.7 to 73.3) |
| + 10% | MED+PSY | 4.8 (19%) | 18.2 (69.9%) | 50.9 (16.2 to 72.3) |
| + 20% | MED+PSY | 4.8 (19%) | 17.8 (68.5%) | 49.5 (14.9 to 71.3) |
| + 30% | MED+PSY | 4.8 (19%) | 17.5 (67.2%) | 48.2 (13.4 to 70.6) |
| + 40% | MED+PSY | 4.8 (19%) | 17.3 (66.5%) | 47.5 (13.0 to 70.0) |
| + 50% | MED+PSY | 4.8 (19%) | 17.1 (65.8%) | 46.8 (12.4 to 69.5) |
| BDI-II (MAR) | IBI-ONLY | 99.3 (14.6%) | 314.4 (43.2%) | 28.6 (22.1 to 35.1) |
| + 10% | IBI-ONLY | 99.3 (14.6%) | 309.1 (42.5%) | 27.9 (21.4 to 34.3) |
| + 20% | IBI-ONLY | 99.3 (14.6%) | 304.2 (41.8%) | 27.2 (20.8 to 33.6) |
| + 30% | IBI-ONLY | 99.3 (14.6%) | 300.9 (41.3%) | 26.7 (20.5 to 33.0) |
| + 40% | IBI-ONLY | 99.3 (14.6%) | 297.9 (40.9%) | 26.3 (20.1 to 32.6) |
| + 50% | IBI-ONLY | 99.3 (14.6%) | 295.4 (40.6%) | 26.0 (19.7 to 32.3) |
| **Note.** Results of the sensitivity analysis. +10% indicates that the scores imputed using multiple imputation for individuals in the INT arm—excluding those already imputed alongside the CAU group—were increased by 10%. Similarly, +20% indicates that the imputed scores used in the primary analysis reported in the manuscript were increased by 20%. This approach simulates a Not Missing at Random (NMAR) scenario, where the imputations may underestimate the symptom burden of individuals in the INT-arm, potentially leading to an overestimation of the between-group effect. The analysis explores the tipping point at which the conclusions change. | | | | |
